# Clinical Advances and Challenges in Targeting KRAS Mutations in Non-Small Cell Lung Cancer

**DOI:** 10.3390/cancers16223885

**Published:** 2024-11-20

**Authors:** Simone E. Dekker, Lei Deng

**Affiliations:** 1 Division of Hematology and Oncology, Department of Medicine, School of Medicine, University of Washington, Seattle, WA 98195, USA; dekker@uw.edu; 2 Clinical Research Division, Fred Hutchinson Cancer Center, Seattle, WA 98109, USA

**Keywords:** KRAS, G12C, lung cancer, non-small cell lung cancer, targeted therapy

## Abstract

KRAS mutation is one of the most common oncogenic drivers in non-small cell lung cancer. Since its discovery about four decades ago, drug development targeting KRAS has been met with countless failures. Recently, KRAS G12C, a subvariant of KRAS, became the first druggable KRAS mutation. The efficacy of the first-generation KRAS inhibitor is modest, but with scientific advancement, KRAS G12C inhibitors with higher potency are on the horizon. Additionally, novel therapeutic approaches targeting other KRAS subvariants are also being explored in clinical trials with encouraging early data. We will review the clinical advances and challenges for patients with KRAS-mutated non-small cell lung cancer, with a focus on small molecule inhibitors.

## Simple Summary

As one of the most common genetic alterations, KRAS mutations play a pivotal role in the growth of lung cancer, particularly non-small cell lung cancer. After 40 years of research, patients with KRAS G12C-mutated lung cancer can now benefit from novel KRAS-targeting drugs. However, the efficacy is far from optimal, but newer experimental drugs showed promising results. It is also likely that other KRAS mutations will likely be druggable in the future.

## 1. Introduction

As one of the most common driver oncogenes in cancers, including non-small cell lung cancers (NSCLCs) [1], KRAS mutation has long been considered as undruggable [2]. Numerous attempts have been made to target it directly or through up- or downstream pathways, but success has not been widely seen. However, scientific and technological advances have moved the needle in recent years. A subvariant, G12C, became the first targetable KRAS mutation with approved drugs. Nevertheless, the efficacy of first-generation KRAS G12C inhibitors is relatively modest compared to the deep and durable response seen in other targeted therapies [3,4] inhibiting EGFR, ALK, ROS-1, etc. [5,6]. In this review article, we discuss the advances and challenges in targeting KRAS mutations in NSCLC with a focus on strategies that are currently in clinical development. Clinical trials were identified on clinicaltrials.gov using the following search criteria: (1) condition/disease: lung cancer; (2) other terms: KRAS; (3) study status: recruiting or active but not recruiting; (4) age: adult (18–64 years old) or older adult (65+); (5) study type: interventional.

## 2. KRAS Mutation in NSCLC

**Figure 1:**
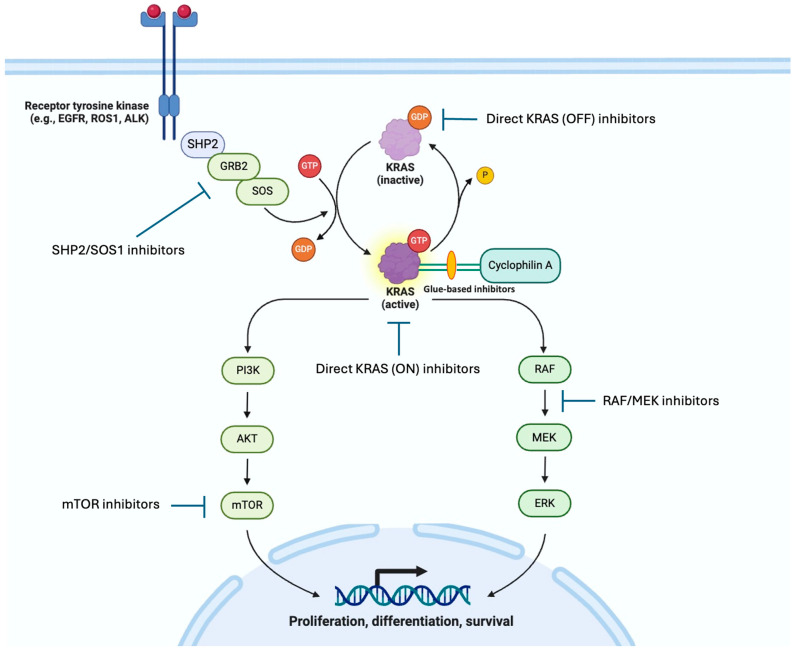
KRAS pathway and targets for treatment.

KRAS is the most common oncogenic driver in lung adenocarcinoma, accounting for about a quarter of patients [7]. It belongs to a large member of plasma-bound protein known as GTPase. When it is bound to GTP, it is in the active state (ON). Then, GTP is hydrolyzed to GDP, an inactive state (OFF). KRAS cycles through ON and OFF states, controlling the downstream effectors, including the MAPK, PI3K, and RALGDS pathways and many others, promoting the growth and survival of cancer cells [8]. The mutated KRAS oncoprotein can keep KRAS in its GTP-bound ON state, driving cancer growth (Figure 1) [9]. Due to a lack of targeted therapy options, KRAS mutation is associated with worse survival compared to other driver oncogenes.

Unlike driver oncogenes like EGFR, ALK, and ROS-1, KRAS-driven NSCLC is more likely to be associated with smoking and sensitivity to immune checkpoint inhibitors [10,11,12,13]. It is also accompanied by co-occurring mutations, which are associated with differing sensitivity not only to immune checkpoint inhibitors and chemotherapy, but also to novel KRAS inhibitors. KRAS-mutated NSCLC with STK11 and/or KEAP1 mutations have been reported to have poorer prognosis in various reports [14]. This may create opportunities for KRAS inhibitors to improve outcomes for those subsets of patients.

KRAS G12C is the most common subvariant in NSCLC, accounting for 30–40% of the KRAS-mutated cases, followed by G12V, G12D, G12A, and G13X [15,16]. In the era of chemotherapy, the KRAS subvariant appears to be similar in terms of prognosis [17]. However, the biological implications of these subvariants may vary. For example, KRAS G12A is preferentially associated with the activated PI3K and MAPK pathways, while G12C/V had activated Ral signaling and decreased Akt activation [18]. KRAS G12D may be associated with enhanced glutathione-mediated detoxification [19]. 

## 3. Previous Attempts in Targeting KRAS in NSCLC

Before the advent of direct KRAS inhibitors, various strategies were explored to target KRAS-driven cancers. RAS protein needs to be lipophilic to be attached to the cellular membrane to function. Therefore, farnesyltransferase, initially considered as the key enzyme in this process, was explored as a target but was unsuccessful [20,21,22]. It was discovered that geranylgeranyl transferase I alternatively allows the membrane location and signal transduction of KRAS. The results of targeting the downstream pathway of KRAS including RAF, MEK, PI3K, and mTOR were also disappointing [9]. SHP2 and SOS1 play a vital role in upstream RTK-mediated KRAS activation [23,24,25,26]. Therefore, the inhibitors of these two molecules were also explored in clinical trials. However, SHP2 agents are now being discontinued by several major pharmaceutical companies, possibly due to unfavorable toxicity profiles [27].

## 4. Direct KRAS G12c Targeting in NSCLC

### 4.1. Approved KRAS G12C Inhibitors

Being the most common KRAS subvariant in NSCLC, KRAS G12C became the first targetable mutation with two US FDA-approved drugs—sotorasib and adagrasib. KRAS G12C cycles through active and inactive stages. In 2013, a new pocket beneath the effector binding switch-II region was reported [28]. A covalent inhibitor binding to this pocket locks the KRAS G12C in the inactive GDP-bound state, thus disrupting downstream Raf signaling. Thus, these first-generation inhibitors are considered as KRAS G12C (OFF) inhibitors, because only the OFF (inactive) state is targeted.

Sotorasib is the first-in-class KRAS G12C-specific covalent (OFF) inhibitor that received regulatory approval [29]. CodeBreak 100 is a single-arm, phase II trial evaluating sotorasib in 126 patients with NSCLC [30]. Eligible patients must have received prior platinum-based chemotherapy and/or anti-PD(L)1 immunotherapy. The objective response rate (ORR) was 37.1% with a median progression-free survival (PFS) of 6.8 months. The median overall survival (OS) was 12.5 months. The treatment discontinuation rate due to adverse events was 7%. FDA made the historic regulatory decision by granting accelerated approval in May 2021, eight years after the initial report of the novel binding pocket identified in 2013. To confirm sotorasib’s efficacy, it was compared head-to-head with docetaxel. The study confirmed sotorasib’s superiority over docetaxel in its primary endpoint, with a statistically significant improvement of median PFS from 4.5 to 5.6 months (HR 0.66). Sotorasib also improved ORR with fewer serious treatment-related adverse events (TRAEs). There was no difference in OS, but the trial was not powered to test OS difference. 

Adagrasib is another first-generation KRAS G12C-specific (OFF) inhibitor that received the FDA’s accelerated approval [31]. KRYSTAL-1 enrolled 116 previously treated patients with an ORR of 42.9% and median PFS at 6.5 months. Most recently, its confirmatory KRYSTAL-12 trial, a head-to-head comparison against docetaxel, was read out [32]. The primary endpoint, PFS, was met with an improvement from 3.8 to 5.5 months (HR 0.58). 

Fulzerasib (GFH925/IBI351) is a KRAS G12C (OFF) inhibitor that has received approval in China. The phase 2 single-arm trial of previously treated patients showed a confirmed ORR of 49% and a median PFS of 9.7 months [33]. 

However, the incremental efficacy over docetaxel appears to be modest and is overshadowed by the durable response when targeting other oncogenic drivers. The FDA called an oncologic drugs advisor committee (ODAC) meeting to discuss CodeBreak 200 data [34]. The FDA held the stance that the small sample size, marginal PFS difference, and concerns about trial conduct integrity may not be interpretable. The ODAC upheld the FDA’s stance overwhelmingly by voting 10-2. There are numerous novel compounds that are in active clinical investigation (Table 1).

### 4.2. Resistance to Direct KRAS G12C (OFF) Inhibitors 

Similarly to other targeted therapies, resistance inevitably develops to KRAS G12C (OFF) inhibitors. Intrinsic resistance mechanisms may involve co-occurring mutations, including KEAP1 [4]. The acquired resistance mechanism largely remains unclear. However, preliminary clinical results suggest that putative resistance mechanisms may be diverse and heterogeneous [35]. Other acquired KRAS subvariants, including KRAS G12X, G13D, Q61H, etc., were reported [36]. Putative bypass resistance mechanisms included MET amplification, a mutation in NRAS, BRAF, and oncogenic fusions, including ALK and RET. Histological transformation to squamous-cell carcinoma was also reported. These putative resistance alterations also co-exist in 41% of the patients, making it challenging to develop a one-size-fits-all strategy to target. Preclinical studies also showed that the newly synthesized KRAS G12C protein upon treatment maintains its ON state, leading to resistance to KRAS G12C OFF inhibitors [37]. Recent studies have demonstrated that Hippo (YAP/TAZ) signaling is a significant contributor to resistance against KRAS G12C inhibitors in vitro and in vivo [38,39]. Combining therapies targeting this pathway could enhance the efficacy of G12C inhibitors in cancer patients.

### 4.3. Frontline Efforts 

The less-than-expected efficiency of monotherapy does not appear to compare favorably to frontline options—immunotherapy and/or platinum doublet [40,41,42]. To move the currently approved KRAS G12C inhibitors to the frontline, a combination would be the natural strategy (Table 2).

Sotorasib combination with immune checkpoint inhibitors (ICIs, pembrolizumab, or atezolizumab) was examined in the CodeBreak 100/101 trial. The concurrent administration of sotorasib and ICI was associated with grade 3 or higher TRAE in 60–79% of the patients, driven by hepatotoxicity (~50%). Although sotorasib lead-in is associated with lower TRAE, the rate remains to be high at 30–53% [43]. This is consistent with a retrospective report that sotorasib after ICI is associated with a higher risk of immune-related toxicity, particularly when ICI exposure was 12 weeks prior to sotorasib [44]. Sotorasib plus platinum doublet chemotherapy was examined in the CodeBreak 101 trial, with an ORR of 65% and median PFS at 10.8 months, in the first-line setting [43]. Currently, CodeBreak 202 is a phase 3 trial to directly combine sotorasib vs. pembrolizumab in combination with platinum doublet chemotherapy in PD-L1 negative patients [45,46].

Adagrasib appears to have more favorable toxicity data when in combination with ICIs. KRYSTAL-7 is a phase 2 clinical trial which showed that treatment-related hepatic events occurred in less than 10% of the patients [47]. The trial also reported an ORR of 49% and 63% among all PD-L1 subgroups and PD-L1 TPS ≥ 50%, respectively. The phase 3 portion of a head-to-head comparison of adagrasib + pembrolizumab vs. pembrolizumab monotherapy is ongoing in patients with PD-L1 TPS ≥ 50%. Of note, the dosage of adagrasib explored in this combination was 400 mg twice daily, lower than the approved monotherapy dosage of 600 mg twice daily.

Fulzerasib and cetuximab combination was explored in previously untreated patients, showing an encouraging ORR of 82%. In patients with low or negative PD-L1 expression, the response was also above 60%, which is not dependent on baseline EGFR expression [48].

### 4.4. Emerging KRAS G12C Inhibitors 

More potent KRAS G12C inhibitors are urgently needed for better efficacy. Divarasib is a novel KRAS G12C (OFF) inhibitor. A phase 1 study showed confirmed ORR of 53%, comparing favorably to currently approved inhibitors [49]. Olomorasib is another promising KRAS G12C (OFF) inhibitor with an ORR of 39% in patients who have experienced prior KRAS G12C inhibitors [50]. Its combination with pembrolizumab also showed an ORR of 77% and 40% in first-line and previously treated cohorts, respectively. Grade 3 or higher TRAEs were reported in 27% of the patients. The permanent discontinuation of olomorasib and pembrolizumab was reported in 3% and 11% of the patients, respectively. Its first-line phase 3 study—SUNRAY-01—is ongoing to directly compare the addition of olomorasib to the standard of care vs. standard of care alone [51].

KRAS G12C (OFF) inhibitors are inherently limited in that only one conformation is targeted; thus, (ON) inhibitors would be ideal to overcome this shortcoming. RMC-6291 is unique in its novel tri-complex mechanism of KRAS G12C targeting. Instead of binding to a pocket opening at OFF conformation, it serves as a molecular glue with cyclophilin A to bind the KRAS protein to block downstream RAS effector signaling. Therefore, it can inhibit both ON and OFF conformation. In a phase 1 study, 57% of the NSCLC patients had a response [52]. The most common Grade 3 TREAE was QTc prolongation, but all patients were able to stay on treatment after dose reduction.

BBO-8520 and FMC-376 are direct KRAS G12C (ON) and (OFF) inhibitors that have the potential to overcome the inherent shortcoming of OFF state-only targeting. Preclinical data showed that BBO-8520 and FMC-376 are more potent than first-generation (OFF) inhibitors in vitro and in vivo [53,54]. Phase 1 clinical trials are ongoing (NCT06244771, NCT06343402).

## 5. Beyond KRAS G12c Only 

Although G12C is the most common KRAS subvariant, the majority (~60%) of KRAS mutations are non-G12C, including G12D/V/R, G13X, etc. These subvariants can also emerge as a secondary resistance mechanism. Thus, to fully drug KRAS mutations, strategies are urgently needed to expand to non-G12C [55]. Table 3, Table 4 and Table 5 summarize the current ongoing clinical trials investigating KRAS inhibitors beyond the G12C subvariant.

ASP3082 is a protein degrader, specifically targeting KRAS G12D. Early phase I pan-tumor data showed an ORR of 33.3% in 12 patients at 300 mg, including prostate cancer, non-small cell lung cancer, and colorectal cancer [56].

Utilizing the same tri-complex mechanism described above, RMC-6236 is a RAS^MULTI^(ON) inhibitor that has activity across all KRAS mutations. In a phase 1 study of KRAS G12X, RMC-6236 resulted in an ORR of 38% in patients with KRAS G12X in previously treated patients, with activity seen across various subvariants [57]. Only 1% of the patients discontinued treatment due to TRAE in the full NSCLC and pancreatic cohorts. RMC-6236 is also being hypothesized to overcome resistance to a single subvariant-directed inhibitor in a phase 1 trial (NCT06040541). Larger phase 3 data of RMC-6236 is eagerly awaited.

There are also other approaches targeting non-G12C KRAS subvariants, including RAS-RAF clamp (NCT05786924), RAF/MEK clamp [58], protein degradation [59], KRAS-SOS1 inhibition [25].

## 6. Beyond KRAS Small Molecule Inhibitors

In addition to small molecule inhibitors, other therapeutic strategies are also being explored. Notably, immunotherapy including vaccine and cellular therapy trials are ongoing (NCT05254184, NCT06253520, NCT06043713, NCT06105021). The efficacy of those strategies is eagerly awaited.

RAS plays a role in metabolic pathways, such as tumor metabolism, glutaminolysis, redox homeostasis, lipid metabolism, and nutrient scavenging. Targeting oncogenic RAS-related metabolism is a potential area for further investigation [60]. This presents a promising avenue for future research, potentially leading to novel translational therapies for KRAS-mutant NSCLC.

## 7. Conclusions and Future Directions

Forty years since the initial discovery of KRAS mutation, numerous publications have highlighted the pivotal role of KRAS mutation in various cancers, including NSCLC. Today, we are finally in an era when a subset of KRAS can be targeted. This success is just the beginning, with more exciting progress and opportunities awaiting ahead.

Academic and industry partners will continue to take on the challenges of (1) making all KRAS subvariants targetable with balanced tolerability and efficacy; (2) frontline KRAS therapeutics strategy; (3) intrinsic and acquired resistance mechanisms; (4) tolerable and rational combination approach.

## Figures and Tables

**Table 1:** Ongoing clinical trials of KRAS G12C small molecule inhibitor monotherapies.

Compound	Mechanism of Action	Clinical Trial Indication	Phase of Study
Sotorasib (AMG510)	KRAS G12C OFF inhibitor	- Compared to docetaxel in previously treated locally advanced or unresectable or metastatic NSCLC (NCT04303780) - Frontline in advanced NSCLC (NCT06582771) - Frontline in stage IV NSCLC (NCT04933695) - Frontline in stage IIA-IIIB(NCT05118854) - In previously treated locally advanced or metastatic NSCLC (NCT05631249) - Stage III unresectable NSCLC ineligible for chemo-radiation therapy (NCT05398094) - In metastatic or recurrent NSCLC (NCT04625647) - Compared to durvalumab in locally advanced NSCLC with MRD (NCT06333678) - Stage III/IV not amenable for curative treatment (NCT05311709)	3 2 2 2 2 2 2 2 2
Adagrasib (MRTX849)	KRAS G12C OFF inhibitor	- Compared to docetaxel in advanced NSCLC (NCT04685135) - First line with or without pembrolizumab (NCT04613596) - In patients who are elderly or have poor performance status (NCT05673187) - In advanced solid tumors (NCT03785249)	3 2 2 1/2
Garsorasib (D-1553)	KRAS G12C OFF inhibitor	- Compared to docetaxel in locally advanced NSCLC in second line (NCT06300177) - In advanced or metastatic NSCLC (NCT05383898)	3 1/2
Divarasib (GDC-6036)	KRAS G12C OFF inhibitor	- Advanced or metastatic disease (NCT06497556) - Advanced or metastatic disease (NCT04449874)	3 1
Opnurasib (JDQ-443)	KRAS G12C OFF inhibitor	- Second line after platinum-based chemotherapy and ICI as monotherapy compared to docetaxel (NCT05132075) - In advanced solid tumors (NCT05358249)	3 1/2
JDQ443	KRAS G12C OFF inhibitor	- As monotherapy compared to docetaxel in stage IIIB/IIIC or metastatic disease (NCT05132075)	3
Glecirasib (JAB-21822)	KRAS G12C OFF inhibitor	- First line for locally advanced or metastatic NSCLC with PDL 1 < 1% or PD-L1 > 1% and STK11 co-mutation (NCT05445843) - In locally advanced or metastatic NSCLC (NCT05276726)	2 1/2
HBI-2438	KRAS G12C OFF	- Advanced solid tumors (NCT05485974)	1
Olomorasib (LY3537982)	KRAS G12C OFF	- In locally advanced unresectable or metastatic NSCLC (NCT04956640)	1/2
FMC-376	KRAS G12C ON and OFF inhibitor	- In locally advanced unresectable or metastatic solid tumors (NCT06244771)	1/2
RMC-6291	Tri-complex (glue-based) mechanism of KRAS G12C targeting	- In advanced solid tumors (NCT05462717)	1

**Table 2:** Ongoing clinical trials of combination therapies with KRAS inhibitors.

Combination Strategy	Compound	Clinical Trial Indication
Combination with Chemo, RT and/or ICI	Adagrasib	- Adagrasib in combination with Nivolumab in the neoadjuvant setting (NCT05472623) - Adagrasib in combination with stereotactic radiosurgery in metastatic NSCLC (NCT06248606) - Adagrasib in combination therapy in advanced NSCLC (NCT05609578) - Adagrasib in combination with small molecule PD-L1 inhibitor INCB099280 in advanced solid tumors
	Olomorasib (LY3537982)	- In combination with Pembrolizumab in first line (NCT04613596) - In combination with the standard of care in untreated advanced NSCLC (NCT06119581)
	Sotorasib	- Platinum doublet combination versus Pembrolizumab platinum doublet combination as frontline therapy in IV or stage IIIB/C nonsquamous NSCLC (NCT05920356) - With stereotactic radiation therapy in patients with advanced NSCLC who have received at least one line of standard medical management (NCT06127940)
	BBO-8520	- In combination with Pembrolizumab in advanced NSCLC (NCT06343402)
	MK-1084	- With pembrolizumab as first-line treatment in metastatic NSCLC in TPS ≥ 50% (NCT06345729)
	Fulzerasib(IBI351)	- In combination with Sintilimab ± chemotherapy in advanced non-squamous NSCLC (NCT05504278)
	Garsorasib (D-1553)-	- In combination therapy in locally advanced or metastatic NSCLC (NCT05492045)
	GFH925	- In combination with Cetuximab in advanced NSCLC (NCT05756153)
	Divarasib (GDC-6036)	- Combined with chemotherapy or ICI in previously untreated, advanced or metastatic NSCLC (NCT05789082)
	RMC-6291 or RMC-6236	- Combined with standard of care in KRAS G12C- or pan-RAS-mutated advanced solid tumors (NCT06162221)
Combination with SHP2/SOS1	Sotorasib	- In combination with SHP2 inhibitor BBP-398 in locally advanced or metastatic subjects after the failure of prior standard therapies (NCT05480865) - In combination with RMC-4630 after the failure of standard treatment (NCT05054725)
	Adagrasib	- In combination with TNO155 in advanced solid tumors (NCT04330664)
	Glecirasib and JAB 3312	- In combination with advanced solid tumors (NCT05288205) and (NCT06416410)
Other combinations	Sotorasib with Ras-Raf-Mek-ERK inhibitor	- In combination with Avutometinib and/or Defactinib in stage IIIB-IV (NCT05074810)
	Sotorasib with proteosome inhibitor	- In combination with Carfilzomib in advanced or metastatic NSCLC (NCT06249282)
	Sotorasib in combination with IL-8 receptor inhibitor	- In combination with Ladarixin in advanced NSCLC (NCT05815173)
	Adagrasib with pan-KRAS inhibitor	- In combination with BI 1701963 for metastatic disease (NCT04975256)
	Adagrasib with mTOR inhibitor	- In combination with Nab-Sirolimus in advanced solid tumors (NCT05840510)
	Adagrasib with PARP inhibitor	- In combination with Olaparib in advanced solid tumors (NCT06130254)
	Adagrasib with RAF/MEK clamp	- In combination with VS-6766 in second line NSCLC (NCT05375994)
	KRAS:KRAS G12C ON inhibitor	- RMC-6291 in combination with RMC-6236 in advanced solid tumors (NCT06128551)

**Table 3:** Ongoing clinical trials of other KRAS single subvariant small molecule inhibitor monotherapies.

Compound	Mechanism of Action	Clinical Trial Indication	Phase of Study
MRTX1133	KRAS G12D inhibitor	- In advanced solid tumors (NCT05737706) - Alone or in combination with adagrasib in advanced solid tumors (NCT05578092)	1/2 1/2
TSN1611	KRAS G12D inhibitor	- In advanced solid tumors (NCT06385925)	1/2
RMC-9805 (RM-036)	KRAS G12D inhibitor	- As monotherapy and in combination with RMC-6263 in solid tumors (NCT06040541)	1
ASP3082	KRAS G12D inhibitor	- In locally advanced or metastatic solid tumors (NCT06364696)	1
QLC1101	KRAS G12D inhibitor	- In advanced solid tumors (NCT06403735)	1
INCB161734	KRAS G12D inhibitor	- In advanced or metastatic solid tumors as a single agent or in combination with other anticancer therapies (NCT06179160)	1

**Table 4:** Ongoing clinical trials of pan-KRAS direct small molecule inhibitor monotherapies.

Compound	Mechanism of Action	Clinical Trial Indication	Phase of Study
RMC-6236	Pan-RAS inhibitor	- In advanced solid tumors (NCT05379985)	1
PF-07934040	Pan-KRAS inhibitor	- Given alone or in combination with advanced solid malignancies (NCT06447662)	1
YL-17231	Pan-KRAS inhibitor	- Advanced solid tumors (NCT06078800)	1

**Table 5:** Other KRAS-targeting strategies.

Compound	Mechanism of Action	Indication
KRAS peptide vaccine	Vaccine	- In combination with Nivolumab and Ipilimumab for stage III/IV unresectable NSCLC (NCT05254184)
Targovax TG-01/Stimulon QS-21	Vaccine	- In combination with Daratumumab and Nivolumab in advanced NSCLC (NCT06015724)
KRAS TCR-Transduced PBL and GRT-C903/GRT-R904	KRAS TCR-transduced PBL in combination with KRAS G12D and G12V vaccine	- Second line in solid tumors (NCT06253520)
FH-A11KRASG12V-TCR	Autologous transgenic T cells expressing high-affinity KRASG12V mutation-specific T cell receptors	- In metastatic solid tumors (NCT06043713)
AFNT-211	Autologous CD8+ and CD4+ engineered T cell receptor T Cell	- In advanced or metastatic solid tumors with KRAS G12V mutation (NCT06105021)
NT-112	KRAS G12D autologous T cell therapy	- Unresectable, advanced, and/or metastatic NSCLC (NCT06218914)
