# Correction: Dekker, S.E.; Deng, L. Clinical Advances and Challenges in Targeting KRAS Mutations in Non-Small Cell Lung Cancer. *Cancers* 2024, *16*, 3885

**DOI:** 10.3390/cancers17010098

**Published:** 2024-12-31

**Authors:** Simone E. Dekker, Lei Deng

**Affiliations:** 1 Division of Hematology and Oncology, Department of Medicine, School of Medicine, University of Washington, Seattle, WA 98195, USA; dekker@uw.edu; 2 Clinical Research Division, Fred Hutchinson Cancer Center, Seattle, WA 98109, USA

## Missing Conflicts of Interest

In the original publication [1], the Conflicts of Interest statement was missing. The Conflicts of Interest are included in this correction as follows:

Conflicts of Interest: L.D. declares the following conflicts of interest: Honoraria from MJH Life Sciences, Precisca; Consulting fees from Bristol-Myers Squibb, Regeneron; Travel from Merck, MJH Life Sciences; Institutional research funding from Bridgebio Oncology Therapeutics.

The authors apologize for any inconvenience caused and state that the scientific conclusions are unaffected. This correction was approved by the Academic Editor. The original publication has also been updated.
